# Optimization of Tunisian *Myrtus communis* L. Essential Oil Extraction by Complete Factorial Experimental Design

**DOI:** 10.3390/metabo15060369

**Published:** 2025-06-03

**Authors:** Rania Zayani, Eya BenSalem, Mariem Khouja, Amani Bouhjar, Mohamed Boussaid, Chokri Messaoud

**Affiliations:** Laboratory of Nanobiotechnologies LR24ES19, Department of Biology, National Institute of Applied Sciences and Technology, Carthage University, B.P. 676, Tunis Cedex 1080, Tunisia; eyabensalem97@gmail.com (E.B.); khouja.mar@gmail.com (M.K.); amanibouhjar95@gmail.com (A.B.); boussaid.med11@gmail.com (M.B.)

**Keywords:** *Myrtus communis*, leaves, essential oil, experimental design, yield, chemical composition, antioxidant activity, response surfaces

## Abstract

**Background:** *Myrtus communis* L. is a typical aromatic species of the Mediterranean basin, whose leaves are rich in essential oil known for its biological properties. **Methods:** The essential oil of Tunisian *Myrtus communis* L. leaves was extracted via hydrodistillation using a Clevenger-type apparatus and optimized using a complete factorial design including three factors with two different modalities and one factor with three modalities, hence the total number of experiments N_total_ = 2^3^ × 3^1^. This optimization concerns the yield, the terpene composition by GC-MS and the antioxidant activity by the two radical scavenging assays, DPPH and ABTS. Four factors were retained, namely, the type of leaf used (dry or fresh sample), the leaf granulometry (whole or ground), the extraction time (1 h 30 min, 2 h 30 min and 3 h 30 min) and the water volume/plant material ratio (1/4 and 1/10). **Results:** The dry and whole leaves, duration 3 h 30 min, and V/M 1/10 modalities gave the best yield of essential oil (0.77%). The optimal contents of the majority of the terpene compounds, 1,8-cineole (37.23%), α-pinene (54.79%), myrtenyl acetate (23.43%) and limonene (17.77%), were recorded using the modalities dry and whole leaves, duration 2 h 30 min, V/M 1/10; dry and ground leaves, duration 1 h 30 min, V/M 1/4; fresh and whole leaves, duration 3 h 30 min, V/M 1/4; and fresh and whole leaves, duration 3 h 30 min, V/M 1/4, respectively. The antioxidant activity of the essential oil of myrtle leaves was optimized for the two DPPH (7.477 mg TE/g EO) with the GDL, duration 3 h 30 min, V/M 1/4 and ABTS assays (14.053 mg TE/g EO) with WDL terms, duration 3 h 30 min, V/M 1/10. **Conclusions:** Optimizing essential oil extraction is of significant interest to the cosmetic, perfumery, and pharmaceutical industries, which are constantly seeking optimal conditions to enhance essential oil yield and to ensure a high concentration of terpenic compounds, valued for their aromatic qualities and diverse biological activities.

## 1. Introduction

*Myrtus communis* L. is an aromatic shrub belonging to the myrtaceae family, widely distributed in the mediterranean area [1,2]. In Tunisia, myrtle is one of the aromatic species of the cork oak forest covering the northwest, Cap Bon and some regions of the Dorsal [3,4]. This species grows naturally across various bioclimatic zones spanning from upper semi-arid to lower humid areas [5]. In addition to its ornamental and aromatic properties [6], *Myrtus communis* L. is widely used in perfumes, cosmetic products [2], medicine, the food industry [7] and mediterranean traditional gastronomy [8]. Myrtle has been reported to treat inflammations [9,10], hypertension [11], diarrhea, diabetes [12,13], ulcers, acne [14], rheumatic pain [15] and Alzheimer’s disease [16].

Myrtle essential oil is highly recommended for its richness in 1,8-cineole, which exerts anti-inflammatory, antioxidant, antimicrobial, and anticancer activities [2,17]. The most commonly used method for extracting essential oil is uncontrolled extraction using standard parameters. Different factors such as extraction time, type and granulometry of leaves, and water volume/plant matter ratio can affect the yield, chemical composition and biological activities of the essential oils. Several recent studies have focused on optimizing the extraction of essential oil from myrtle leaves using experimental design methods. The aim is to maximize both yield and chemical composition for the essential oil industry while minimizing the number of experiments conducted. Kaya et al. explored this approach in their research [18]. Similarly, Zermane et al. applied response surface methodology to optimize the extraction process for Algerian myrtle leaves [19]. In another study, Ammar et al. used a 2^4^ complete factorial design to enhance the yield of essential oil from Tunisian myrtle leaves [4].

The aim of our study is to optimize the operating conditions for the extraction of myrtle leaves’ essential oil via hydrodistillation using a complete factorial experimental design. This approach was selected because it allows the evaluation of all possible factor combinations. Moreover, the number of factors considered in our study is not large enough to require the use of an orthogonal fractional design.

The selected factors are type of leaf used (dry or fresh sample), leaf granulometry (whole or ground), water to plant material ratio (1/10 or 1/4) and extraction time (1 h 30 min, 2 h 30 min, 3 h 30 min). Response surfaces methodology was also elaborated to support the results of the experimental design, indicating the optimal values for the yield of essential oil from myrtle leaves, its major terpene composition and the antioxidant activity measured by DPPH and ABTS radical scavenging assays.

## 2. Materials and Methods

### 2.1. Experimental Material

The plant material consisted of *Myrtus communis* L. leaves collected in March 2020 from Korbous, a locality situated in the Cap Bon region (northeast of Tunisia) belonging to the sub-humid bioclimatic zone with an altitude of 400 m and characterized by clay–sandstone soil.

The material was divided into two portions; one portion was preserved fresh in the dark at −20 °C until further use, while the other portion was shade-dried under ambient conditions for two weeks before storage. Both fresh and dried leaves were subjected to hydrodistillation for the extraction of essential oils, which were subsequently stored under appropriate conditions for analysis.

### 2.2. Relative Water Content

Plant water status was evaluated using the relative water content (RWC, %), following the method described by Yamasaki and Dillenburg [20]. This method involves determining the percentage of water present in excised leaves. Leaf blades were excised at the base and immediately weighed to record the fresh weight (FW). The leaves were then immersed in distilled water and kept in the dark for 24 h to reach full turgor, after which they were weighed to obtain the turgid weight (TW). Finally, the samples were oven-dried at 60 °C for 48 h to determine the dry weight (DW).

The relative water content is expressed as a percentage, and it is given by the following formula:

RWC = [(FW − DW)/(TW − DW)] × 100(1)

### 2.3. Extraction Methods of Myrtle Essential Oil

Essential oils from myrtle (*Myrtus communis* L.) leaves were extracted via hydrodistillation using a Clevenger-type apparatus. The obtained essential oils were dried over anhydrous sodium sulphate and stored in sterile tubes at 4 °C until analysis. Optimization of the extraction process was conducted using a complete factorial experimental design 2^3^ × 3^1^.

The evaluated responses included essential oil yield, chemical composition, and antioxidant activity, which were determined using two radical scavenging assays, DPPH and ABTS. The four selected factors, chosen based on previous studies [4,21,22], were type of leaf used, leaf granulometry, water to plant material ratio, and extraction time.

Details of the studied parameters, including their levels and symbols, are summarized in Table S1 (see Supplementary Material).

### 2.4. Multi-Level Factorial Experimental Design

A multi-level, multi-response factorial design was employed to optimize the efficiency of essential oil extraction from myrtle (*Myrtus communis* L.) leaves. Two factors were fixed: the type of leaf used and their granulometry. The remaining two factors, extraction time and the water to plant material ratio (V/M), were varied at two levels.

This optimization process classified all hydrodistillations into four distinct blocks: block A (fresh, whole leaves), block B (dry, ground leaves), block C (fresh, ground leaves), and block D (dry, whole leaves). Details of the classification are presented in Table S2.

### 2.5. Determination of Essential Oil Yield

The yield was estimated based on the mass of essential oil obtained relative to the mass of fresh or dried plant material, whether whole or ground. It is expressed as a percentage and calculated using the following formula:

Yield (%) = 100 × (Quantity of essential oil obtained/Quantity of plant material)(2)

### 2.6. Antioxidant Activity

The in vitro antioxidant activity of essential oils was assessed through DPPH and ABTS radical scavenging assays.

The free DPPH radical scavenging activity was determined using the methodology outlined by Doshi et al. [23]. When DPPH^•^ interacts with a hydrogen-donating antioxidant compound, it is reduced [24]. In brief, 50 μL of essential oils diluted in methanol were added to 950 μL of freshly prepared methanolic DPPH solution (60 μM). The resulting mixture was vortexed and kept at room temperature for 30 min in the dark. Subsequently, absorbance was measured at 517 nm against the corresponding blank, with a control consisting of 50 μL methanol and 950 μL of DPPH solution. Each determination was performed in triplicate, and the results were expressed as milligrams of Trolox equivalent per gram of essential oil (mg TE/g EO).

The ABTS radical scavenging assay followed the procedure by Khouja et al. [25]. It is applicable for both lipophilic and hydrophilic compounds [24]. Specifically, 10 μL of diluted essential oils were mixed with 990 μL of the ABTS solution. After 5 min incubation in the dark, the residual absorbance of the ABTS radical was measured at 734 nm. Each determination was conducted in triplicate, and the results were expressed as milligrams of Trolox equivalent per gram of essential oil (mg TE/g EO).

Radical scavenging activity was estimated as follows:

Inhibition (%) = 100 × [(A_0_ − A_1_)/A_0_] (3)

where A_0_ and A_1_ represent the absorbance of the control and the absorbance of the sample, respectively.

### 2.7. Chemical Composition

The chemical composition of essential oils was determined through GC-FID and GC-MS analysis following the procedure outlined by Aissi et al. [26]. GC-FID analyses were carried out using an HP-5MS capillary column. The oven temperature was programmed from 60 to 240° C at 4 °C/min. The flame ionization detector and the injector temperatures were 280 °C and 250 °C, respectively. The helium was used as carrier gas (flow = 0.8 mL/min). GC–MS analyses were performed with a gas chromatograph (Agilent 7890A) (Agilent Technologies, Inc., Wilmington, NC, USA), equipped with a HP-5MS capillary column (30 m × 0.25 mm; 0.25 µm film thickness) and associated with a mass selective detector (Agilent 5975C inter MSD) (Agilent Technologies, Inc., Wilmington, NC, USA). The flow of the carrier gas (helium) was 0.8 mL/min. The oven temperature was programmed from 60 to 240 °C at 4 °C/min. The injector temperature was maintained at 250 °C. Temperatures of the quadrupole and the source were 150 and 230 °C, respectively. The mass scan ranged from 50 to 550 *m*/*z* at 70 eV. For each sample, 1 µL was injected.

Terpenic compounds were identified by comparing their retention times with authentic standards analyzed under the same chromatographic conditions. Additionally, identification involved comparing retention indices with literature values and co-injecting essential oils with available authentic standards. Mass spectra of terpenic compounds were compared with those stored in NIST-08 and W8N08 libraries for further confirmation.

The quantification was achieved by using the compounds’ percentages determined from their GC-FID peak areas without correction factors.

### 2.8. Statistical Analysis

All results are presented as mean ± standard deviation (SD). Variations in essential oil yield, composition, and antioxidant activities among samples were analyzed using one-way Analysis of Variance (ANOVA) followed by Duncan’s multiple range test, performed with SAS software (version 9.1.3).

To evaluate the asymmetric complete factorial design, ANOVA and Student’s test were applied to assess the main effects of individual factors and their interactions on each response variable. For each response from the multi-level experimental design, the coefficient of determination (R^2^), the model equation, and optimal conditions represented as response surface plots were determined using variance analysis with Statgraphics Centurion software (version 19).

Statgraphics Centurion software (version 19) was further employed for multi-response optimization, enabling the comparison of factor modalities and determination of optimal values for each response block. This process identified the most effective extraction factor combinations for maximizing myrtle essential oil yield, composition, and antioxidant activity based on the defined desired outcomes.

## 3. Results

### 3.1. Relative Water Content

The relative water content (RWC) of myrtle (*Myrtus communis* L.) leaves was determined by measuring their fresh weight (FW), turgid weight (TW), and dry weight (DW). The fresh weight of the leaves, recorded immediately after collection, was 100 g. Following a 24 h incubation in water under dark conditions, the turgid weight was measured at 110 g. The dry weight, obtained after drying the leaves in an oven at 60 °C for 48 h, was 71 g. The relative water content expressed as a percentage is 74.35%.

### 3.2. Essential Oil Yield

The various extraction of essential oils from myrtle leaves resulted in significantly different yields (*p* < 0.05), ranging from 0.3% ± 0.005 to 0.77% ± 0.006, as shown in Table 1. The lowest yield was observed during the tenth hydrodistillation (H10) using fresh and ground leaves, with an extraction time of 1 h 30 and a water to plant material ratio of 1/4. Conversely, the highest yield among all extractions (H15) was obtained using dry and whole leaves for 3 h 30, with a water to plant material ratio of 1/10 with a yield equal to 0.77% ± 0.006.

#### 3.2.1. Analysis of Factors’ Effects on Yield

**Figure 1:**
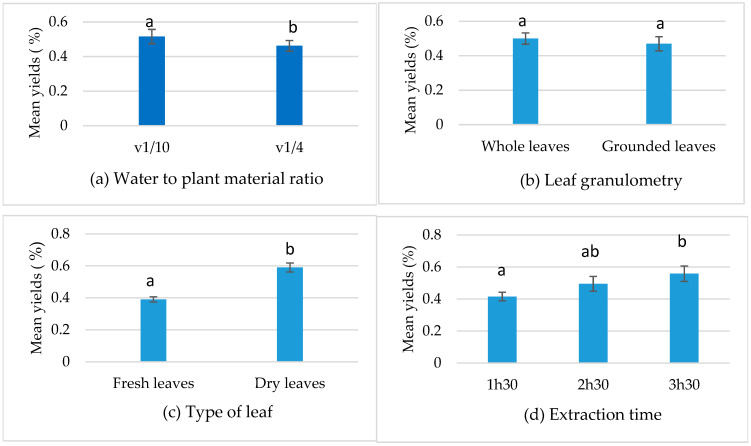
Factors’ effects on yield. (**a**) Water to plant material ratio. (**b**) Leaf granulometry. (**c**) Type of leaf. (**d**) Extraction time. Means followed by different letters are significantly different (*p* (ANOVA test) < 0.05).

The contribution of each factor was evaluated after accounting for the effects of other parameters. Statistical analysis revealed a highly significant effect (*p* ≤ 0.01) for most of the selected factors (Table 2 and Figure 1a,c,d), except for the leaf granulometry factor, which showed no significant variation (*p* > 0.05) between whole and ground leaves in terms of essential oil yield, as shown in Table 2 and Figure 1b.

#### 3.2.2. Effect of Two-Factor Interactions on Yield

The interactions between pairs of factors affecting the yield of myrtle essential oil were analyzed and are summarized in Table S3. Three interactions were identified as highly significant (*p* ≤ 0.01): the interaction between type of leaf used and leaf granulometry (AB), the interaction between type of leaf used and the water to plant material ratio (AC), and the interaction between type of leaf used and extraction time (AD). The other interactions (BC, BD and CD) were identified as non-significant (*p* > 0.05)

### 3.3. Antioxidant Activity

The antioxidant activity of the 24 essential oil samples from *Myrtus communis* L., assessed using DPPH and ABTS radical scavenging assays and expressed as the reduction of free radical concentration by the essential oil, showed significant differences among the samples (*p* < 0.05), as shown in Table 3. Antioxidant activity increased proportionally with the duration of hydrodistillation, with the highest activity observed after 3 h 30 min. Indeed, the highest antioxidant activity was observed at H24 (GDL, extraction time: 3 h 30 min, V/M: 1/4) with a value of 7.477 mg TE/g E.O for DPPH scavenging assay and at H15 (WDL, extraction time: 3 h 30 min, V/M: 1/10) with a value of 14.053 mg TE/g E.O for ABTS assay (Table 3).

However, despite the optimal activity observed in both assays, the overall antioxidant activity of myrtle essential oils remained moderate and not highly significant.

#### 3.3.1. Analysis of the Effects of Factors on Antioxidant Activity

**Figure 2:**
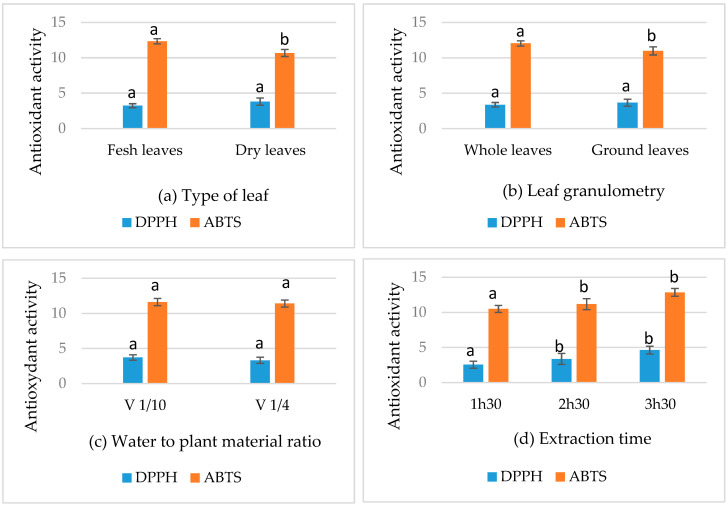
Effect of factors on the anti-radical activity. (**a**) Type of leaf. (**b**) Leaf granulometry. (**c**) Water to plant material ratio. (**d**) Extraction time. Means followed by different letters are significantly different (*p* (ANOVA test) < 0.05).

The effect of each factor studied on the antioxidant activity of myrtle essential oils showed that the anti-radical activity evaluated by the DPPH assay is highly dependent on the extraction time of the essential oil (*p* ≤ 0.01) (Table 4, Figure 2d). Indeed, the optimal activity for this test was observed in the H24, where the duration of the distillation was 3 h 30 min. However, the three other factors (type of leaf used, leaf granulometry and V/M ratio) showed no significant effect on DPPH radical scavenging assay (*p* > 0.05), as demonstrated in Table 4 and Figure 2a–c.

Unlike the DPPH assay, Table 5 shows that the antioxidant activity determined by the ABTS anti-radical assay is significantly influenced by most of the factors selected (*p* < 0.01) (Figure 2a,b,d), except for the parameter “water to plant material ratio” (*p* > 0.05) (Figure 2c).

#### 3.3.2. Effect of Two-Factor Interactions on the Antioxidant Activity

The analysis of variance of the interaction of two factors on the two anti-radical assays was carried out (Tables S4 and S5). For DPPH assay, only the AC, AD and BC interactions were considered significant (0.01 < *p* ≤ 0.05). The ABTS test showed a significant effect by the combinations AB, AC and BC. The other interactions developed were estimated to be non-significant (*p* > 0.05).

### 3.4. Chemical Composition of Myrtle Essential Oil

The chemical composition of essential oils from various extractions was determined using gas chromatography coupled with mass spectrometry (GC-MS). To better evaluate the effect of the experimental design’ factors used, only the four major compounds, α-pinene, 1,8-cineole, limonene, and myrtenyl acetate, were analyzed, as shown in Table 6. The various extractions showed a significantly different terpenic contents for the four major compounds (*p* < 0.05).

The essential oils exhibited a predominance of α-pinene and 1,8-cineole, with α-pinene reaching 54.79% in sample H22 (dry and ground leaves; extraction time: 1 h 30 min; V/M ratio: 1/4) and 1,8-cineole peaking at 37.23% in sample H14 (dry and whole leaves; extraction time: 2 h 30 min; V/M ratio: 1/10).

Significant variations were observed in the percentages of myrtenyl acetate and limonene across the extraction conditions. Myrtenyl acetate ranged from 2.67% in sample H22 to 23.04% in sample H6, where the conditions were fresh and whole leaves; extraction time: 3 h 30 min; and V/M ratio: 1/4. Limonene content varied between 8.32% in sample H18 (dry and whole leaves; extraction time: 3 h 30 min; V/M: 1/4) and 17.77% in sample H6.

These findings suggest that the selection of hydrodistillation factor modalities can be tailored based on the desired terpene composition (Table 7). For instance, an essential oil enriched in 1,8-cineole can be obtained using whole and dry myrtle leaves without grinding, a hydrodistillation duration of 2 h 30 min, and a water to plant material ratio of 1/10.

#### 3.4.1. Analysis of the Effects of Factors on Chemical Composition

##### Analysis of the Effects of Factors on 1,8-Cineole Content

**Figure 3:**
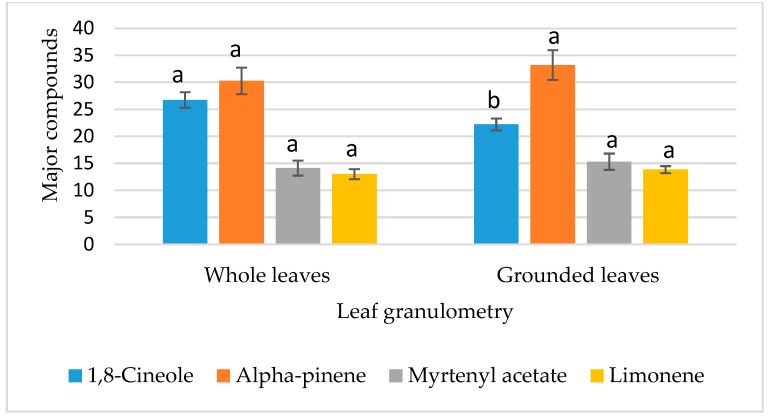
Effect of leaf granulometry on the contents of major compounds. Means followed by different letters are significantly different (*p* (ANOVA test) < 0.05).

**Figure 4:**
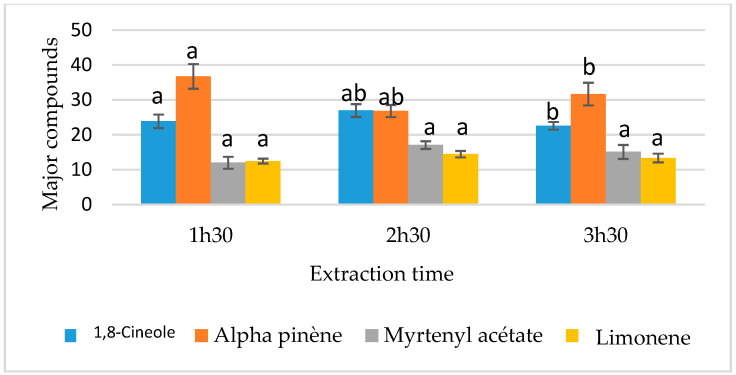
Effect of extraction time on the contents of major compounds. Means followed by different letters are significantly different (*p* (ANOVA test) < 0.05).

**Figure 5:**
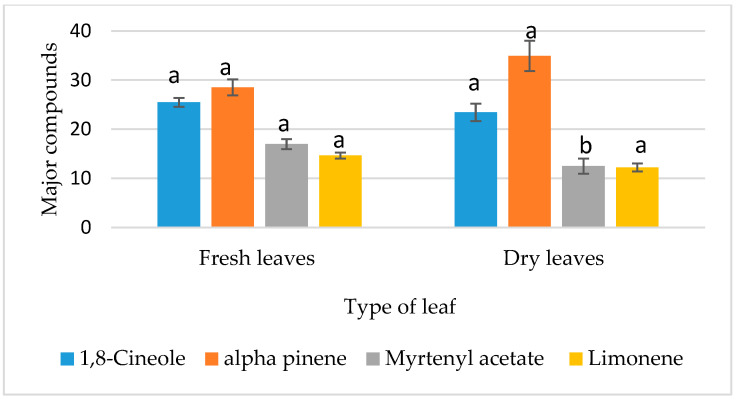
Effect of type of leaf used on the contents of major compounds. Means followed by different letters are significantly different (*p* (ANOVA test) < 0.05).

**Figure 6:**
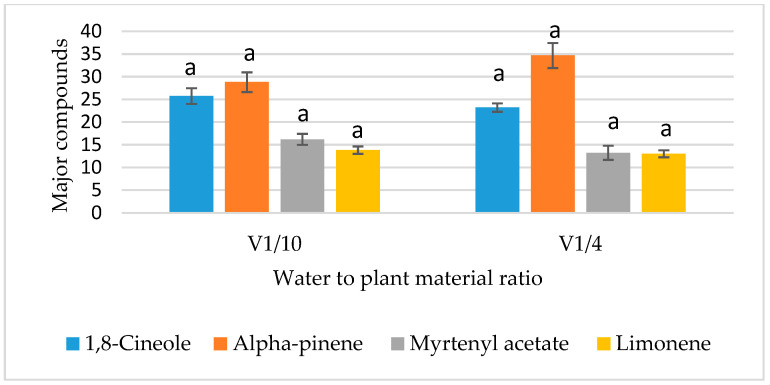
Effect of water to plant material ratio on the contents of major compounds. Means followed by different letters are significantly different (*p* (ANOVA test) < 0.05).

The effects of the four studied factors on the 1,8-cineole content are summarized in Table 8. Among these factors, only “leaf granulometry” and “extraction time” demonstrated a highly significant impact (*p* ≤ 0.01) and a significant effect (0.01 < *p* ≤ 0.05), respectively, on 1,8-cineole content. The results indicated a higher 1,8-cineole value while using whole leaves (Figure 3) and an extraction time of 2 h 30 min (Figure 4). Conversely, the remaining parameters tested (type of leaf used and V/M ratio) did not exhibit a statistically significant effect on 1,8-cineole content (Table 8, Figure 5 and Figure 6).

##### Analysis of the Effects of Factors on α-Pinene Content

The α-pinene content is significantly affected by the extraction time (0.01 < *p* ≤ 0.05). The highest α-pinene content (54.79% ± 0.015) was observed with an extraction duration of 1 h 30 min (Figure 5). In contrast, leaf granulometry, along with the other factors tested, did not have a significant influence (*p* > 0.05) on the α-pinene percentage in the chemical composition of myrtle essential oil (Table 9, Figure 3, Figure 4 and Figure 6).

##### Analysis of the Effects of Factors on Myrtenyl Acetate Content

The chemical composition of the various hydrodistillations revealed that the myrtenyl acetate content is significantly influenced by the type of leaf used (*p* < 0.05) (Table 10). Analysis of the variance for the different extraction conditions indicated that fresh leaves tend to generate a higher myrtenyl acetate content compared to dry leaves, as shown in Figure 3. The three other factors showed no significant effect (*p* > 0.05), as presented in Table 10 and illustrated in Figure 4, Figure 5 and Figure 6.

##### Analysis of the Effects of Factors on the Content of Limonene

In contrast to the other major compounds analyzed, the variance analysis of limonene percentage in the chemical composition of the essential oils revealed that none of the selected factors significantly affected its content (*p* > 0.05) as shown in Table 11. As a result, all factor modalities in the experimental design reveal the same limonene content (Figure 3, Figure 4, Figure 5 and Figure 6). This suggests that limonene content is independent of the parameters used in the hydrodistillation process.

#### 3.4.2. Effect of Interactions Between the Two Factors on 1,8-Cineole Content

An analysis of variance was conducted to examine the interactions between pairs of factors on the chemical composition of myrtle essential oils. Only the interaction between leaf granulometry and the water to plant material ratio (BC) significantly affected the 1,8-cineole content (*p* = 0.0135), as shown in Table S6. All other factor combinations were considered insignificant for 1,8-cineole as well as for the other terpenes (*p* > 0.05).

### 3.5. Analysis of the Multi-Level Experimental Design

#### 3.5.1. Block A: Fresh and Whole Leaves

The combinations of modalities for the extraction time and water to plant material ratio factors that aim to maximize the responses studied are presented in Table 12. Regarding the analysis of this first block, only the yield and antioxidant activity, as assessed by DPPH and ABTS assays, exhibited an R-squared (R^2^) value greater than 90%. (R^2^ = 98.57% for yield, R^2^ = 99.48% for DPPH assay and R^2^ = 99.86% for ABTS assay)

The 3D graphs illustrate the combined effects of the operating factors on the essential oil yield obtained under different experimental conditions of extraction time and water to plant material ratio.

**Figure 7:**
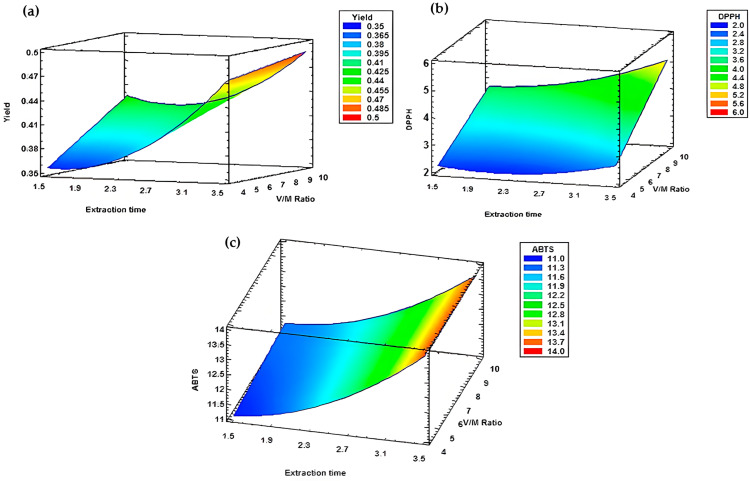
The response surfaces retained from block A. (**a**) Yield. (**b**) DPPH assay. (**c**) ABTS assay.

The analysis of the response surfaces (Figure 7) for the appropriate models from block A revealed that extraction time and the water to plant material (V/M) ratio significantly affect both yield and antioxidant activities, as assessed by DPPH and ABTS assays. The optimal extraction time for all three desired responses was 3 h 30 min, with a V/M ratio of 1/10 for yield (Figure 7a) and DPPH activity (Figure 7b). However, for radical scavenging activity measured by ABTS assay, the optimal ratio was 1/4 (Figure 7c).

##### Block A Multi-Response Optimization

Only the responses with adequate models (R^2^ > 90%) were selected for multi-response optimization. For block A, where “type of leaf used” and “leaf granulometry” were fixed as fresh and whole, the three responses’ yield, DPPH, and ABTS were considered to optimize the multi-level response. The optimal factor proportions were determined by correlating the centered reduced variables with the actual values of each factor.

Statgraphics software provided the optimal modalities to maximize the desired responses under the fixed conditions of fresh and whole leaves, an extraction time of 3 h 30 min and a V/M ratio of 1/10 (Table 13).

#### 3.5.2. Block B: Dry and Ground Leaves

**Figure 8:**
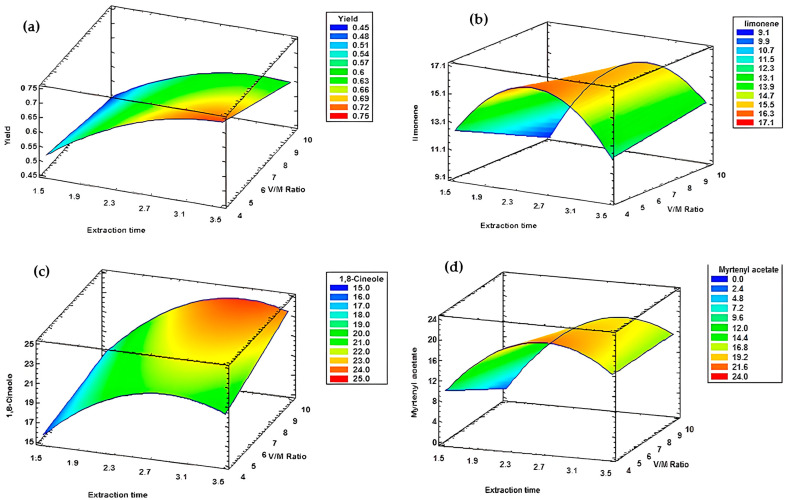

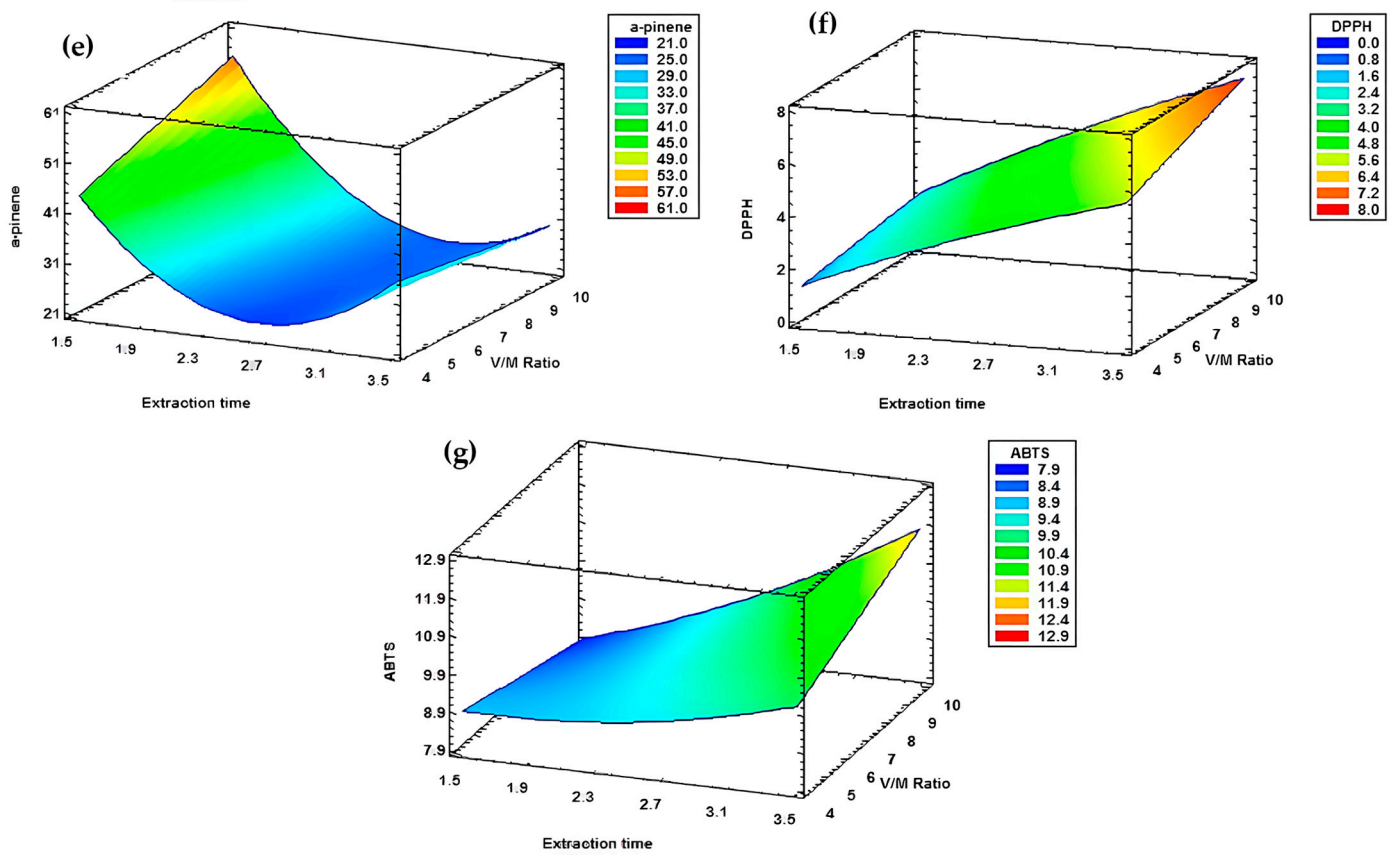
Response surfaces of block B. (**a**) Yield. (**b**) Limonene. (**c**) 1,8-cineole. (**d**) Myrtenyl acetate. (**e**) Alpha-pinene. (**f**) DPPH assay. (**g**) ABTS assay.

For the second block, where type of leaf used and leaf granulometry were fixed as dry and ground, all response models exhibited an R^2^ superior than 90%. For this reason, seven regression model equations and seven response surfaces were generated, as presented in Table 14 and Figure 8.

The results demonstrated that both extraction time and the water to plant material (V/M) ratio significantly influence the responses. More specifically, an increase in extraction time and a decrease in the V/M ratio led to a substantial decrease in yield (Figure 8a). Conversely, a decrease in extraction time and an increase in the V/M ratio resulted in a reduction in α-pinene content (Figure 8e). Additionally, simultaneous increases in both extraction time and V/M ratio maximized radical scavenging activity, as measured by both DPPH (Figure 8f) and ABTS (Figure 8g) assays, and enhanced 1,8-cineole content (Figure 8c).

For myrtenyl acetate and limonene, the response surfaces exhibited a curved pattern (Figure 8b,d), indicating that optimal extraction occurred around 2 h 30 min with a V/M ratio of 1/4. Under these conditions, the maximum contents reached 21.00% for myrtenyl acetate and 16.31% for limonene.

##### Block B Multi-Response Optimization

By combining all responses from block B, where dry and ground leaves were held as constant parameters, the optimal extraction time and V/M ratio were identified. Multi-level optimization of this second extraction block revealed that an extraction time of 3 h 30 min, coupled with a V/M ratio of 1/9.9, provided the best conditions to maximize the desired responses during hydrodistillation with dry and ground leaves (Table 15).

#### 3.5.3. Block C: Fresh and Ground Leaves

The analysis of responses from block C, where fresh and ground leaves were fixed, was conducted using Statgraphics software in the same manner as the other blocks. The responses exhibiting an R-squared (R^2^) value greater than 90% in this block were yield (R^2^ = 99.62%), myrtenyl acetate content (R^2^ = 99.69%), and antioxidant activity, as measured by DPPH (R^2^ = 92.47%) and ABTS (R^2^ = 97.31%) radical scavenging assays (Table 16).

**Figure 9:**
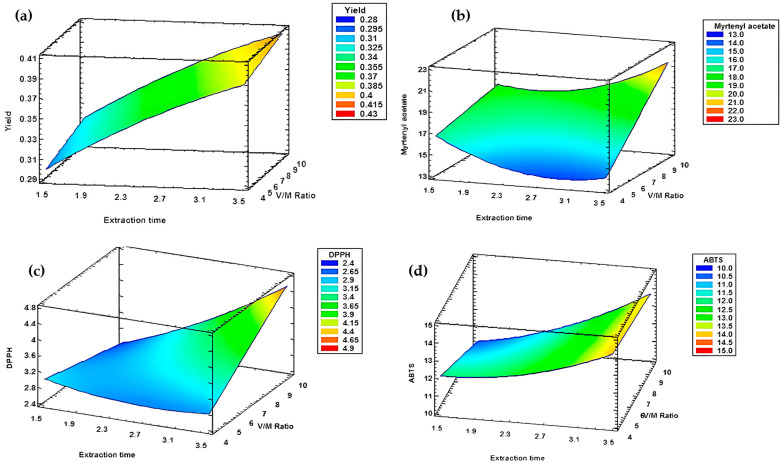
Response surfaces of block C. (**a**) Yield. (**b**) Myrtenyl acetate. (**c**) DPPH assay. (**d**) ABTS assay.

All selected responses showed a significant correlation with the variability of the factors. The optimal conditions for this block were an extraction time of 3 h 30 min and a V/M ratio of 1/10 (Figure 9a–c), except the ABTS response, for which the optimal ratio was 1/4 (Figure 9d)

##### Block C Multi-Response Optimization

The multi-level analysis revealed that the optimal settings were 3 h 30 min for extraction time and a V/M ratio of 1/10, similar to the first block where fresh and whole leaves were used as fixed parameters. This result closely aligns with that of the second block, with only a slight variation in the V/M ratio (Table 17).

#### 3.5.4. Block D: Dry and Whole Leaves

The final analysis was conducted on Block D, where the qualitative factors, type of leaf used, and leaf granulometry were fixed as dry and whole leaves. The variation in the other two factors, extraction time and V/M ratio, across all desired responses showed R-squared correlation coefficients exceeding 90% for the majority of the results, except for limonene content (R^2^ = 52.96%) and radical scavenging activity as measured by DPPH assay (R^2^ = 89.68%) (Table 18).

**Figure 10:**
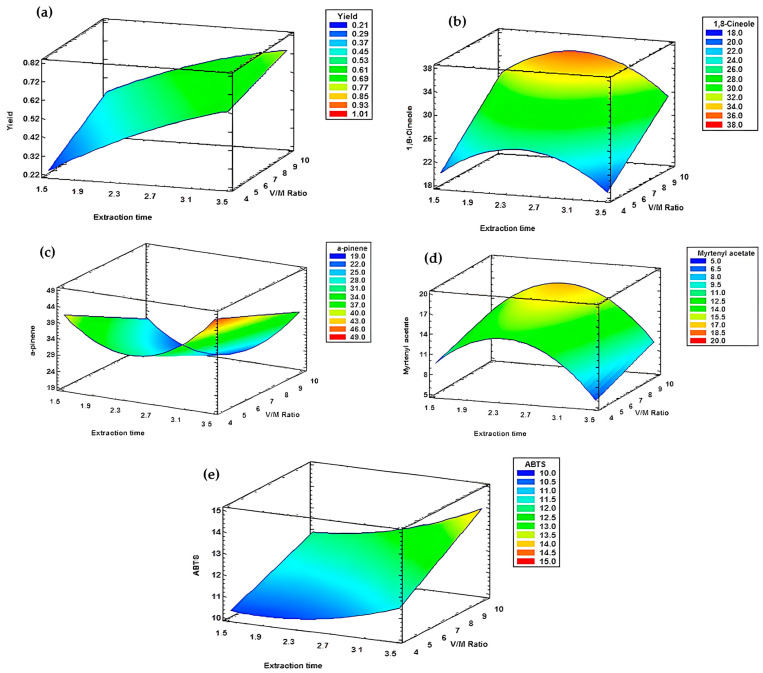
Response surfaces from block D. (**a**) Yield. (**b**) 1,8-cineole. (**c**) Alpha-pinene. (**d**) Myrtenyl acetate. (**e**) ABTS assay.

The response surfaces demonstrated that both extraction time and V/M ratio significantly influenced the selected responses. All the graphs below (Figure 10) display non-flat shapes, indicating the substantial impact of altering the settings of these two factors. For yield and ABTS assay, the optimal conditions were the longest extraction time (3 h 30 min) and the highest V/M ratio (1/10), which provided the most favorable settings to achieve the optimal value for these responses (Figure 10a,e). Conversely, the optimal V/M ratio for α-pinene was ¼ (Figure 10c).

For 1,8-cineole and myrtenyl acetate, the response surfaces demonstrate a curved pattern (Figure 10b,d) indicating thar optimal extraction occurred around 2h30min with a V/M ratio of 1/10. 

##### Block D Multi-Response Optimization

The combination of the five responses analyzed through multi-response optimization allowed the determination of the average optimal values for extraction time and the water to plant material ratio. By fixing the type of leaf used and leaf granulometry factors as dry and whole leaves and varying the other two parameters, it was concluded that the optimal conditions for maximizing the desired responses are an extraction time of 3 h 20 min and a V/M ratio of 1/10 (Table 19).

## 4. Discussion

The yield of essential oils extracted from *Myrtus communis* L. leaves ranged from 0.3% to 0.77%, indicating significant variability (Table 1). This variation may be attributed to the different factors of the experimental design used. These findings are consistent with previously published results, including a yield of 0.635% reported by Ammar et al. [4], 0.61% by Aidi Wannes et al. [3] and 0.57% by Moura et al. [27]. During this study, the complete factorial design using four distinct factors, type of leaf used (dry or fresh sample), leaf granulometry (whole or ground), extraction time (1 h 30 min, 2 h 30 min, and 3 h 30 min), and the water to plant material ratio (1/4 and 1/10), revealed a significant difference among the studied responses. The optimal essential oil yield was recorded in H15, which corresponded to the conditions WDL, 3 h 30 min, and 1/10. These parameters provided the highest essential oil yield. Indeed, using whole, dried leaves minimizes potential molecule loss during grinding and eliminates the water content present in fresh leaves. Moreover, a longer extraction time and a higher water to plant material ratio facilitate the release of the essential oil contained in the leaves. These results are consistent with the findings of Kaya et al. [18] and Ghasemi et al. [28] who demonstrated that extraction time significantly influences the essential oil yield of *Myrtus communis* L. leaves extracted by steam distillation and supercritical fluid, respectively. However, the work of Kaya et al. [18] demonstrated that using ground myrtle leaves resulted in a higher essential oil yield when applying steam distillation. This indicates that the optimal conditions for the studied factors may vary depending on the extraction method used and enables industries utilizing myrtle essential oil, such as the cosmetic and perfumery industries, to identify the optimal parameters for maximizing yield for each extraction method employed.

The study of the antioxidant activity of myrtle leaves’ essential oil showed moderate activity and significant differences among the samples (Table 3). The highest activity using DPPH radical scavenging assay was recorded in sample H24 (GDL, 3 h 30 min, 1/4), with the value of 7.477 ± 0.016 mg Eq Trolox/g E.O, whereas the most significant activity using ABTS assay was registered in H15 (WDL, 3 h 30 min, 1/10), with the value of 14.053 ± 0.011 mg Eq Trolox/g E.O. These radical-scavenging properties can be attributed to the terpenic composition obtained under these hydrodistillation conditions. Previous studies conducted on the same plant also revealed moderate activity expressed in IC_50_ through the two anti-radical assays, DPPH and ABTS [9,29,30,31].

The chemical composition of the essential oil derived from myrtle leaves was analyzed using gas chromatography-mass spectrometry (GC-MS). Similar to previous studies, myrtle essential oil represents a diverse array of terpenic compounds depending on geographical area, plant parts and plant phenological stage [31]. In our study, the 24 samples of essential oil obtained from Tunisian myrtle leaves collected during the vegetative stage show a chemical profile with four predominant constituents, comprising over 50% of the total composition, which are α-pinene, 1,8-cineole, myrtenyl acetate, and limonene (Table 6). The factorial design applied in this study, aiming to provide valuable insights for facilitating the selection of the best conditions to obtain the desired terpenic composition, revealed significant variations in the chemical composition of essential oils extracted from myrtle leaves. The optimal contents of these terpenes were recorded under different hydrodistillation conditions. α-pinene, a widely applied terpene in the flavor and fragrance industry due to its characteristic woody aroma [32,33,34], was identified as the predominant compound in the majority of the hydrodistillation samples, with an optimal percentage of 54.79% observed in H22 (GDL, 1 h 30 min, 1/4). This significant content of alpha-pinene is considerably higher than that recorded in previous studies of Tunisian [35,36] and mediterranean [2,37,38] myrtle leaves’ essential oil. These findings differ from those reported by Messaoud et al. and Brada et al., who recorded α-pinene contents ranging from 1.7% to 11.4% in myrtle berries’ essential oil [39,40]. In short-duration hydrodistillations, this hydrocarbon monoterpene exhibited the highest concentrations within the chemical profile, which may be due to the delayed release of other terpenes requiring longer extraction times. However, exceptions were observed in samples H2, H6, H7, H13, and especially H14 (WDL, 2 h 30 min, 1/10), where 1,8-cineole, valued for its distinctive eucalyptus-like aroma [33], was the principal terpenic constituent with a value of 37.23%. This content is higher than that reported by Mansour et al. (9.9%) [36] and Bazzali et al. [41] for myrtle leaves’ essential oil as well as Messaoud et al. (16.3%) [39] and Brada et al. (11.4%) [40] for myrtle berries’ essential oil. Our results are in agreement with the findings of Aidi Wannes et al. (32.84%) [3] and Dhouibi et al. (37%) [35].

As for myrtenyl acetate and limonene, two terpenes characterized by their woody-mentholated and citrus-like aromas, respectively [33], the 24 hydrodistillations performed recorded significantly different concentrations for both compounds, depending on the extraction modalities used. Their highest concentrations were recorded in H6 (WFL, 3 h 30 min, 1/4). Regarding myrtenyl acetate, several studies have shown its absence in myrtle leaves’ essential oil such as Dhouibi et al. [35], Mohamadi et al. [7] and Yarahmadi et al. [42], as well as myrtle berries’ essential oil [39,40]. However, the chemical profile attained by Moura et al. [27] and Mansour et al. [36] shows similar results to ours. Comparing the chemical composition of our study to those obtained in previous works, it is noticeable that the fourth major component, limonene, is generally higher than those of Smeti et al. (7.52%) [43], Bahadirli et al. (6.16%) [44] and Dejam and Farahmand (0.74%) [45] for the myrtle leaves and Messaoud et al. (5.4%) [39] for myrtle berries’ essential oil.

The study reported by Karaoğul and Alma showed highly variable terpenic contents between two different methods of extraction using hydrodistillation and solvent-free microwave extraction across different pine species [46]. This significant variation highlights the impact not only of the extraction factors but also of the extraction method used on the terpenic composition of essential oils.

The various statistical analyses as well as the multi-level optimization carried out on the four blocks of experiments by fixing the two factors (Type of leaf used and leaf granulometry) and varying the other two showed that most of the blocks suggest the modalities of extraction time 3 h 30 min and ratio V/M 1/10 for the optimization of the hydrodistillation of the essential oil of *Myrtus communis* L. leaves. This optimization concerns seven distinct responses, namely, the yield, the chemical composition and, more precisely, the contents of 1,8-cineole, α-pinene, limonene and myrtenyl acetate, and the antioxidant activity through the two anti-radical assays, DPPH and ABTS.

The optimization of extraction conditions will not only provide a better understanding of the factors influencing the quality and quantity of the essential oil but also offer more efficient and cost-effective extraction methods. This work can be transferred to the agro-food, cosmetic, and pharmaceutical industries, which seek to exploit the flavored aroma of the different terpenic compounds as well as the biological properties of myrtle essential oil, particularly its antioxidant potential. The technological transfer resulting from the implementation of these improved methods on an industrial scale will facilitate the integration of this natural product into commercial formulations and enhance the competitiveness of companies in the field of cosmetics and natural health products.

Although the complete factorial design used in our study explored four key factors, other variable such as distillation temperature can impact the efficiency of hydrodistillation. Excessive heat can cause the degradation of volatile compounds, leading to a loss of yield, while insufficient heat may result in incomplete volatilization of certain terpenes, reducing overall extraction efficiency.

## 5. Conclusions

This study aims to optimize the extraction of essential oil from myrtle leaves by maximizing the essential oil yield and the terpenic compounds contents and enhancing antioxidant activity. This optimization could be beneficial for the cosmetic, perfumery, and pharmaceutical industries, which continuously seek optimal conditions not only to maximize essential oil yield but also to achieve high contents of terpenic compounds. These compounds are widely used for their characteristic aromas and biological properties like antioxidant activity.

A complete factorial design was applied to evaluate the effect of four factors on the hydrodistillation process of *Myrtus communis* L. leaves. This classical statistical experimental design was complemented by the response surface methodology to enhance the extraction process, allowing for the selection of optimal factor modalities for each response.

The highest essential oil yield and antioxidant activity via ABTS assay were obtained using whole and dry leaves, an extraction time of 3 h 30 min, and a water to plant material ratio (V/M) of 1/4. For the highest antioxidant activity via DPPH radical scavenging assay, the best conditions were ground and dry leaves, an extraction time of 3 h 30 min, and a V/M of 1/4.

Regarding the essential oil composition, the highest α-pinene content was recorded with ground and dry leaves, an extraction time of 1 h 30 min, and a V/M of 1/4. The optimal 1,8-cineole content was achieved using whole and dry leaves, an extraction time of 2 h 30 min, and a V/M of 1/10. Finally, the highest myrtenyl acetate and limonene contents were obtained with whole and fresh leaves, an extraction time of 3 h 30 min, and a V/M of 1/4.

This research serves as a starting point for further studies and should continue by optimizing the extraction of essential oil from myrtle leaves for other biological activities, such as anti-inflammatory activity, tyrosinase and collagenase inhibition activities, as well as optimizing for the plant’s different stages of maturation to obtain the best desired responses.

## Figures and Tables

**Table 1:** Yield of different extractions of myrtle essential oil.

Experiment	Hydrodistillation Modalities	E.O Yield (%)
H1	WFL, Duration 1 h 30 min, V/M 1/10	0.42 ^ij^ ± 0.003
H2	WFL, Duration 2 h 30 min, V/M 1/10	0.43 ^i^ ± 0.003
H3	WFL, Duration 3 h 30 min, V/M 1/10	0.49 ^gh^ ± 0.003
H4	WFL, Duration 1 h 30 min, V/M 1/4	0.36 ^mn^ ± 0.005
H5	WFL, Duration 2 h 30 min, V/M 1/4	0.37 ^lm^ ± 0.003
H6	WFL, Duration 3 h 30 min, V/M 1/4	0.48 ^gh^ ± 0.006
H7	GFL, Duration 1 h 30 min, V/M 1/10	0.32 ^O^ ± 0.003
H8	GFL, Duration 2 h 30 min, V/M 1/10	0.37 ^m^ ± 0.005
H9	GFL, Duration 3 h 30 min, V/M 1/10	0.41 ^jk^ ± 0.003
H10	GFL, Duration 1 h 30 min, V/M 1/4	0.3 ^po^ ± 0.005
H11	GFL, Duration 2 h 30 min, V/M 1/4	0.35 ^n^ ± 0.000
H12	GFL, Duration 3 h 30 min, V/M 1/4	0.39 ^lk^ ± 0.005
H13	WDL, Duration 1 h 30 min, V/M 1/10	0.47 ^h^ ± 0.003
H14	WDL, Duration 2 h 30 min, V/M 1/10	0.61 ^d^ ± 0.008
H15	WDL, Duration 3 h 30 min, V/M 1/10	0.77 ^a^ ± 0.006
H16	WDL, Duration 1 h 30 min, V/M 1/4	0.47 ^q^ ± 0.014
H17	WDL, Duration 2 h 30 min, V/M 1/4	0.49 ^gh^ ± 0.005
H18	WDL, Duration 3 h 30 min, V/M 1/4	0.61 ^d^ ± 0.003
H19	GDL, Duration 1 h 30 min, V/M 1/10	0.51 ^f^ ± 0.012
H20	GDL, Duration 2 h 30 min, V/M 1/10	0.69 ^c^ ± 0.005
H21	GDL, Duration 3 h 30 min, V/M 1/10	0.72 ^b^ ± 0.011
H22	GDL, Duration 1 h 30 min, V/M 1/4	0.47 ^h^ ± 0.006
H23	GDL, Duration 2 h 30 min, V/M 1/4	0.57 ^e^ ± 0
H24	GDL, Duration 3 h 30 min, V/M 1/4	0.61 ^d^ ± 0.003

Values are presented as means ± SD (*n* = 3). Means followed by different letters within the same column are significantly different (*p* (ANOVA test) < 0.05).

**Table 2:** Analysis of variance of factors’ effects on yield.

Source	Sum of Squares	DF	Mean Square	F	Probability
Main effects					
A: Type of leaf	0.175	1	0.175	115.00	0 **
B: Leaf granulometry	0.000	1	0.000	0.00	0.959 ns
C: Water to plant material ratio	0.039	1	0.039	25.75	0.001 **
D: Extraction time	0.122	2	0.061	39.95	0 **

ns: Non-significant (*p* (Student’s Test) > 0.05); **: Highly significant (*p* (Student’s Test) ≤ 0.01).

**Table 3:** Antioxidant activity of myrtle essential oil.

Experiment	DPPH (mg TE/g E.O)	ABTS (mg TE/g E.O)
H1	3.788 ^fg^ ± 0.011	11.330 ^h^ ± 0.058
H2	3.988 ^f^ ± 0.012	12.033 ^f^ ± 0.118
H3	5.133 ^d^ ± 0.006	13.706 ^c^ ± 0.097
H4	2.166 ^n^ ± 0.012	11.080 ^i^ ± 0.096
H5	2.230 ^mn^ ± 0.010	11.796 ^g^ ± 0.029
H6	2.573 ^lm^ ± 0.012	13.830 ^bc^ ± 0.022
H7	2.623 ^kl^ ± 0.040	10.480 ^j^ ± 0.097
H8	3.076 ^hij^ ± 0.020	11.217 ^hi^ ± 0.040
H9	4.710 ^e^ ± 0.014	13.950 ^ab^ ± 0.049
H10	2.853 ^jkl^ ± 0.054	11.896 ^fg^ ± 0.029
H11	3.000 ^ijk^ ± 0.011	12.836 ^d^ ± 0.022
H12	2.640 ^kl^ ± 0.592	13.930 ^ab^ ± 0.011
H13	2.614 ^kl^ ± 0.027	11.986 ^fg^ ± 0.022
H14	2.743 ^jkl^ ± 0.031	12.506 ^e^ ± 0.029
H15	5.637 ^c^ ± 0.057	14.053 ^a^ ± 0.011
H16	2.997 ^ijk^ ± 0.054	10.283 ^k^ ± 0.022
H17	3.227 ^hi^ ± 0.005	10.466 ^j^ ± 0.017
H18	3.427 ^gh^ ± 0.025	11.384 ^h^ ± 0.047
H19	1.340 ^o^ ± 0	8.780 ^n^ ± 0.019
H20	3.610 ^g^ ± 0.027	9.350 ^m^ ± 0.009
H21	5.453 ^cd^ ± 0.024	9.930 ^l^ ± 0.009
H22	2.108 ^n^ ± 0.036	8.101 ^o^ ± 0.043
H23	5.100 ^d^ ± 0	9.334 ^m^ ± 0
H24	7.477 ^a^ ± 0.016	11.986 ^fg^ ± 0.205

Values are presented as means ± SD (*n* = 3). Means followed by different letters within the same column are significantly different (*p* (ANOVA test) < 0.05).

**Table 4:** Effect of factors on the inhibitory activity of DPPH.

Source	Sum of Squares	DF	Mean Square	F	Probability
Main effects					
A: Type of leaf	2.007	1	2.007	3.50	0.094 ns
B: Leaf granulometry	0.493	1	0.493	0.86	0.378 ns
C: V/M ratio	1.000	1	1.000	1.75	0.219 ns
D: Extraction time	17.429	2	8.714	15.20	0.001 **

ns: Non-significant (*p* (Student’s Test) > 0.05); **: Highly significant (*p* (Student’s Test) ≤ 0.01).

**Table 5:** Effect of factors on the inhibitory activity of ABTS.

Source	Sum of Squares	DF	Mean square	F	Probability
Main effects					
A: Type of leaf	16.517	1	16.517	36.73	0 **
B: Leaf granulometry	6.668	1	6.668	14.83	0.004 **
C: V/M ratio	0.246	1	0.246	0.55	0.478 ns
D: Extraction time	23.407	2	11.703	26.03	0 **

ns: Non-significant (*p* (Student’s Test) > 0.05); **: Highly significant (*p* (Student’s Test) ≤ 0.01).

**Table 6:** Content (%) of the four major compounds in myrtle essential oils.

Hydrodistillation	α-Pinene	Limonene	1,8-Cineole	Myrtenyl-Acetate
	RT:5.479	RT:7.911	RT: 7.973	RT: 17.375
	RI: 936	RI: 1033	RI: 1035	RI: 1333
H1	30.66 ^m^ ± 0.008	12.06 ^n^ ± 0.020	27.84 ^f^ ± 0.023	14.11 ^l^ ± 0.020
H2	21.02 ^w^ ± 0.015	17.05 ^b^ ± 0.026	31.15 ^b^ ± 0.046	21.33 ^c^ ± 0.017
H3	27.92 ^r^ ± 0.012	16.5 ^e^ ± 0.014	24.14 ^l^ ± 0.066	15.22 ^j^ ± 0.014
H4	31.74 ^j^ ± 0.008	13.11 ^l^ ± 0.017	27.19 ^h^ ± 0.020	12.63 ^o^ ± 0.018
H5	30.04 ^n^ ± 0.020	11.68 ^q^ ± 0.008	26.10 ^i^ ± 0.031	14.62 ^k^ ± 0.015
H6	18.55 ^y^ ± 0.017	17.77 ^a^ ± 0.014	23.66 ^m^ ± 0.031	23.04 ^a^ ± 0.029
H7	26.42 ^t^ ± 0.014	16.06 ^f^ ± 0.027	27.43 ^g^ ± 0.017	18.04 ^e^ ± 0.029
H8	31.23 ^k^ ± 0.014	15.05 ^g^ ± 0.028	24.84 ^j^ ± 0.011	18.52 ^d^ ± 0.015
H9	22.27 ^u^ ± 0.012	16.92 ^c^ ± 0.018	20.18 ^q^ ± 0.014	21.35 ^c^ ± 0.023
H10	36.49 ^f^ ± 0.012	13.79 ^i^ ± 0.020	24.30 ^k^ ± 0.012	16.82 ^f^ ± 0.014
H11	29.89 ^o^ ± 0.003	13.49 ^k^ ± 0.012	28.27 ^e^ ± 0.012	13.65 ^m^ ± 0.020
H12	36.23 ^g^ ± 0.014	12.14 ^m^ ± 0.024	20.64 ^p^ ± 0.008	14.08 ^l^ ± 0.017
H13	26.74 ^s^ ± 0.020	11.83 ^g^ ± 0.017	30.72 ^c^ ± 0.015	13.02 ^n^ ± 0.017
H14	20.51 ^x^ ± 0.018	9.92 ^s^ ± 0.017	37.23 ^a^ ± 0.006	16.32 ^g^ ± 0.014
H15	35.96 ^i^ ± 0.014	9.75 ^t^ ± 0.017	28.76 ^d^ ± 0.008	9.91 ^p^ ± 0.011
H16	41.55 ^e^ ± 0.017	11.27 ^r^ ± 0.012	20.72 ^o^ ± 0.003	9.25 ^r^ ± 0.024
H17	31.09 ^l^ ± 0.037	16.76 ^d^ ± 0.018	23.67 ^m^ ± 0.005	14.65 ^k^ ± 0.017
H18	47.48 ^b^ ± 0.020	8.32 ^v^ ± 0.017	19.48 ^r^ ± 0.008	5.47 ^s^ ± 0.017
H19	45.53 ^d^ ± 0.016	12.12 ^m^ ± 0.014	15.67 ^t^ ± 0.006	9.36 ^q^ ± 0.023
H20	21.25 ^v^ ± 0.017	16.91 ^c^ ± 0.017	20.69 ^po^ ± 0.003	21.75 ^b^ ± 0.017
H21	36.06 ^h^ ± 0.023	11.77 ^p^ ± 0.014	20.15 ^p^ ± 0.012	15.50 ^i^ ± 0.008
H22	54.79 ^a^ ± 0.015	9.46 ^u^ ± 0.023	17.02 ^s^ ± 0.015	2.67 ^t^ ± 0.020
H23	29.51 ^p^ ± 0.015	14.69 ^h^ ± 0.020	23.70 ^m^ ± 0.008	15.63 ^h^ ± 0.018
H24	28.84 ^q^ ± 0.023	13.63 ^j^ ± 0.014	23.54 ^n^ ± 0.01	16.27 ^g^ ± 0.014

RT: Retention time; RI: Retention indices relative to n-alkanes (C9–C24) on HP-5MS column. Values are presented as means ± SD (*n* = 3). Means followed by different letters within the same column are significantly different (*p* (ANOVA test) < 0.05).

**Table 7:** Optimal extraction modalities for major terpenic compounds.

Experience	Hydrodistillation Modalities			Desired Compound	Optimum Value
	Type and granulometry of leaves	Extraction time	V/M		
H14	Whole and dry leaves	2 h 30 min	1/10	1,8-cineole	37.23 ± 0.006
H22	Ground and dry leaves	1 h 30 min	1/4	α-pinene	54.79 ± 0.015
H6	Whole and fresh leaves	3 h 30 min	1/4	Myrtenyl acetate	23.43 ± 0.029
H6	Whole and fresh leaves	3 h 30 min	1/4	Limonene	17.77 ± 0.014

**Table 8:** Analysis of the effects of factors on 1,8-cineole content.

Source	Sum of Squares	DF	Mean Square	F	Probability
Main effects					
A: Type of leaf	24.827	1	24.827	2.47	0.150 ns
B: Leaf granulometry	122.447	1	122.447	12.19	0.007 **
C: V/M ratio	38.786	1	38.786	3.86	0.081 ns
D: Extraction time	81.395	2	50.697	5.05	0.05 *

ns: Non-significant (*p* (Student’s Test) > 0.05); *: Significant (0.01 < *p* (Student’s Test) ≤ 0.05); ** Highly significant (*p* (Student’s Test) ≤ 0.01).

**Table 9:** Analysis of the effects of factors on α-pinene content.

Source	Sum of Squares	DF	Mean Square	F	Probability
Main effects					
A: Type of leaf	246.016	1	246.016	4.07	0.074 ns
B: Leaf granulometry	51.744	1	51.744	0.86	0.379 ns
C: V/M ratio	207.917	1	207.917	3.44	0.096 ns
D: Extraction time	393.893	2	196.947	3.26	0.05 *

ns: Non-significant (*p* (Student’s Test) > 0.05); *: Significant (0.01 < *p* (Student’s Test) ≤ 0.05).

**Table 10:** Analysis of the effects of factors on myrtenyl acetate content.

Source	Sum of Squares	DF	Mean Square	F	Probability
Main effects					
A: Type of leaf	119.751	1	119.751	6.45	0.032 *
B: Leaf granulometry	8.225	1	8.225	0.44	0.522 ns
C: V/M ratio	52.955	1	52.955	2.85	0.126 ns
D: Extraction time	104.621	2	52.311	2.82	0.112 ns

ns: Non-significant (*p* (Student’s Test) > 0.05); *: Significant (0.01 < *p* (Student’s Test) ≤ 0.05).

**Table 11:** Analysis of the effects of factors on limonene content.

Source	Sum of Squares	DF	Mean Square	F	Probability
Main effects					
A: Type of leaf	35.527	1	35.527	4.85	0.055 ns
B: Leaf Granulometry	4.150	1	4.150	0.57	0.471 ns
C: V/M ratio	4.002	1	4.002	0.55	0.479 ns
D: Extraction time	15.719	2	7.860	1.07	0.382 ns

ns: Non-significant (*p* (Student’s Test) > 0.05).

**Table 12:** Adequacy of block A models.

Response	Model Adequacy (R^2^)	Model Equation	Optimum	Optimal Value
Yield	98.57%	Yield = 0.392153 − 0.110833 X + 0.0176389 Y + 0.0375 X^2^ − 0.00416667 XY	X = 3 h 30 Y = 1/10	0.5
% 1,8-cineole	82.15%	--------	--------	-------
% α-pinene	62.08%	--------	--------	-------
%Myrtenyl acetate	64.32%	--------	--------	-------
% Limonene	61.35%	--------	--------	-------
DPPH	99.48%	DPPH = 3.00833 − 1.65833 X + 0.134167 Y + 0.31 X^2^ + 0.0783333 XY	X = 3 h 30 Y = 1/10	5.08
ABTS	99.86%	ABTS = 11.6297 − 1.38917 X + 0.0976389 Y + 0.5775 X^2^ − 0.0308333 XY	X = 3 h 30 Y = 1/4	13.80

X = Extraction time, Y = V/M ratio, ------ = Model equation not retained (R^2^ < 90%).

**Table 13:** Optimal modalities of block A.

Response	Optimal Value	Optimal Modalities
Yield	0.49	X = 3 h 30 min Y = 1/10
DPPH	5.09	
ABTS	13.74	

X = Extraction time, Y = V/M ratio.

**Table 14:** Adequacy of block B models.

Response	Model Adequacy (R^2^)	Model Equation	Optimum	Optimal Value
Yield	98.60%	Yield = 0.0860417 + 0.390833 X − 0.000416667 Y − 0.0525 X^2^ − 0.00583333 XY	X = 3 h 30 Y = 1/4	0.73
% 1,8-cineole	99.75%	Cin = −4.11757 + 17.0808 X + 0.00652778 Y − 3.1025 X^2^ + 0.169167 XY	X = 3 h Y = 1/10	24.34
% α-pinene	97.69%	Pin = 119.01 − 78.8667 X + 4.00556 Y + 15.925 X^2^ − 1.37333 XY	X = 1 h 30 Y = 1/10	55.99
% Myrtenyl acetate	98.49%	Myrt = −26.4912 + 39.3083 X − 2.2225 Y − 7.745 X^2^ + 0.621667 XY	X = 2 h 42 Y = 1/4	21.00
% Limonene	96.72%	Lim = −4.12611 + 18.5683 X − 1.11056 Y − 4.05 X^2^ + 0.376667 XY	X = 2 h 29 Y = 1/4	16.31
DPPH	99.99%	DPPH = −3.02583 + 2.935 X − 0.0241667 Y − 0.26 X^2^ + 0.105 XY	X = 3 h 30 Y = 1/10	7.50
ABTS	98.16%	ABTS = 11.8768 − 2.11333 X − 0.494722 Y + 0.355 X^2^ + 0.228333 XY	X = 3 h 30 Y = 1/10	11.87

X = Extraction time, Y = V/M ratio.

**Table 15:** Optimal modalities of block B.

Response	Optimum	Optimal Modalities
Yield	0.60	X = 3 h 30 min Y = 1/9.9
Limonene	13.32	
1,8-cineole	23.62	
Myrtenyl acetate	15.75	
α-pinene	30.08	
DPPH	7.48	
ABTS	11.86	

X = Extraction time, Y = V/M ratio.

**Table 16:** Adequacy of block C models.

Response	Model Adequacy (R^2^)	Model Equation	Optimum	Optimal Value
Yield	99.62%	Yield = 0.220833 + 0.045 X + 0.00333333 Y	X = 3 h 30 Y = 1/10	0.41
% 1,8-cineole	86.57%	--------	-------	---------
% α-pinene	61.38%	--------	-------	--------
% Myrtenyl acetate	99.69%	Myrt = 28.653 − 10.8242 X − 0.518194 Y + 1.4875 X^2^ + 0.504167 XY	X = 3 h 30 Y = 1/10	21.45
% Limonene	80.04%	--------	---------	---------
DPPH	92.47%	DPPH = 5.5441 − 1.7175 X − 0.375139 Y + 0.1675 X^2^ + 0.1925 XY	X = 3 h 30 Y = 1/10	4.57
ABTS	97.31%	ABTS = 15.2331 − 2.165 X − 0.467222 Y + 0.54 X^2^ + 0.12 XY	X = 3 h 30 Y = 1/4	14.08

X = Extraction time, Y = V/M ratio, ------ = Model equation not retained (R^2^ < 90%).

**Table 17:** Optimal modalities of block C.

Response	Optimum	Optimal Modalities
Yield	0.41	X = 3 h 30 min Y = 1/10
Myrtenyl acetate	21.45	
DPPH	4.57	
ABTS	13.80	

X = Extraction time, Y = V/M ratio.

**Table 18:** Adequacy of block D models.

Response	Model Adequacy (R^2^)	Model Equation	Optimum	Optimal Value
Yield	98.83%	Yield = −0.401458 + 0.3825 X + 0.0470833 Y − 0.0325 X^2^ − 0.0075 XY	X = 3 h 30 Y = 1/10	0.75
% 1,8-cineole	97.76%	Cin = −15.9336 + 27.27 X + 1.97444 Y − 5.53 X^2^ − 0.06 XY	X = 2 h 25 Y = 1/10	35.97
% α-pinene	99.54%	Pin = 111.311 − 58.7942 X − 2.73597 Y + 12.1325 X^2^ + 0.274167 XY	X = 3 h 30 Y = 1/4	47.05
% Myrtenyl acetate	97.50%	Myrt = −21.0236 + 28.24 X + 0.411944 Y − 6.07 X^2^ + 0.055 XY	X = 2 h 23 Y = 1/10	17.23
% Limonene	52.96%	----------	---------	---------
DPPH	89.68%	----------	---------	---------
ABTS	99.93%	ABTS = 11.1795 − 1.98583 X + 0.155694 Y + 0.4425 X^2^ + 0.0808333 XY	X = 3 h 30 Y = 1/10	14.04

X = Extraction time, Y = V/M ratio, ------ = Model equation not retained (R^2^ < 90%).

**Table 19:** Optimal modalities of block D.

Response	Optimum	Optimal Modalities
Yield	0.73	X = 3 h 20 min Y = 1/10
1,8-cineole	31.16	
α-pinene	32.18	
Myrtenyl acetate	11.50	
ABTS	13.75	

X = Extraction time, Y = V/M ratio.

## Data Availability

The data that support the findings of this study are available from the corresponding author upon reasonable request.

## Supplementary Materials

The following supporting information can be downloaded at: https://www.mdpi.com/article/10.3390/metabo15060369/s1, Table S1: Factors and modalities of experimental design; Table S2: Asymmetrical experiment matrix of myrtle essential oil extraction; Table S3: Interaction effect of the two factors on yield; Table S4: Effect of two factors’ interactions on DPPH assay; Table S5: Effect of two factors’ interactions on ABTS assay; Table S6: Effect of interactions of the two factors on the 1,8-cineole content; Figure S1: GC-MS Chromatogram.

## Abbreviations

ABTS	2,2′-azino-bis(3-ethylbenzothiazoline-6-sulfonic acid)
DPPH	2,2-diphényl 1-picrylhydrazyle
DW	dry weight
E.O	Essential oil
FW	Fresh weight
GC-MS	Gas chromatography coupled with mass spectrometry
GDL	Ground dry leaves
GFL	Ground fresh Leaves
RWC	Relative water content
TE	Trolox equivalent
TW	Turgid weight
V/M	Water to plant material ratio
WDL	Whole dry Leaves
WFL	Whole fresh Leaves
