# Supplementary material for: Optimization of Tunisian Myrtus communis L. Essential Oil Extraction by Complete Factorial Experimental Design
Source: Metabolites. 2025 Jun 3;15(6):369. doi: 10.3390/metabo15060369 (PMC12195216; doi:10.3390/metabo15060369)
Supplement: Supplementary file 1 [file metabolites-15-00369-s001.zip › metabolites-3481586-supplementary.pdf]

Abbreviation table.

Abbreviations	
ABTS	2,2'-azino-bis(3-ethylbenzothiazoline-6-sulfonic acid)
DPPH	2,2-diphényl 1-picrylhydrazyle
DW	dry weight
E.O	Essential oil
FW	Fresh weight
GC-MS	Gas chromatography coupled with mass spectrometry
GDL	Ground dry leaves
GFL	Ground fresh Leaves
RWC	Relative water content
TE	Trolox equivalent
TW	Turgid weight
V/M	Water to plant material ratio
WDL	Whole dry Leaves
WFL	Whole fresh Leaves

Table S1. Factors and modalities of experimental design.

Parameter	Code	-1	0	1
Type of leaf	X1	Fresh (F)	---	Dry (D)
Leaf granulometry	X2	Whole (W)	---	Ground (G)
Water to plant material ratio (V/M)	X3	1/4	---	1/10
Extraction time	X4	1h30	2h30	3h30

Table S2. Asymmetrical experiment matrix of myrtle essential oil extraction.

Bloc	Experience	X1	X2	X3	X4	Yield of EO	Composition	Antioxidant activity
Bloc A	H1	-1	-1	+1	-1	Y1	C1	A1
	H2	-1	-1	+1	0	Y2	C2	A2
	H3	-1	-1	+1	+1	Y3	C3	A3
	H4	-1	-1	-1	-1	Y4	C4	A4
	H5	-1	-1	-1	0	Y5	C5	A5
	H6	-1	-1	-1	+1	Y6	C6	A6
Bloc C	H7	-1	+1	+1	-1	Y7	C7	A7
	H8	-1	+1	+1	0	Y8	C8	A8
	H9	-1	+1	+1	+1	Y9	C9	A9
	H10	-1	+1	-1	-1	Y10	C10	A10
	H11	-1	+1	-1	0	Y11	C11	A11
	H12	-1	+1	-1	+1	Y12	C12	A12
Bloc D	H13	+1	-1	+1	-1	Y13	C13	A13
	H14	+1	-1	+1	0	Y14	C14	A14

	H15	+1	-1	+1	+1	Y15	C15	A15
	H16	+1	-1	-1	-1	Y16	C16	A16
	H17	+1	-1	-1	0	Y17	C17	A17
	H18	+1	-1	-1	+1	Y18	C18	A18
Bloc B	H19	+1	+1	+1	-1	Y19	C19	A19
	H20	+1	+1	+1	0	Y20	C20	A20
	H21	+1	+1	+1	+1	Y21	C21	A21
	H22	+1	+1	-1	-1	Y22	C22	A22
	H23	+1	+1	-1	0	Y23	C23	A23
	H24	+1	+1	-1	+1	Y24	C24	A24

Tables S3. Interaction effect of the two factors on yield.

Source	Sum of squares	DF	Mean square	F	Probability
INTERACTIONS					
AB: Type of leaf and leaf granulometry	0.027	1	0.027	17.95	0.0022**
AC: Type of leaf and V/M ratio	0.015	1	0.0145	9.53	0.0130**
AD: Type of leaf and Extraction time	0.031	2	0.016	10.18	0.0049**
BC: Leaf granulometry and V/M ratio	0.004	1	0.004	2.63	0.1393 ns
BD: Leaf granulometry and Extraction time	0.008	2	0.004	2.66	0.1235 ns
CD: V/M ratio and Extraction time	0.000	2	0.000	0.10	0.9047 ns

Table S4. Effect of two factors' interactions on DPPH assay.

Source	Sum of squares	DF	Mean square	F	Probability
INTERACTIONS					
AB: Type of leaf and leaf granulometry	1.224	1	1.224	2.14	0.1780 ns
AC: Type of leaf and V/M ratio	4.86	1	4.86	8.48	0.0173*
AD: Type of leaf and Extraction time	5.418	2	2.709	4.73	0.0395*
BC: Leaf granulometry and V/M ratio	3.889	1	3.888	6.78	0.0285*
BD: Leaf granulometry and Extraction time	2.739	2	1.370	2.39	0.1471 ns
CD: V/M ratio and Extraction time	1.900	2	0.950	1.66	0.2439 ns

Table S5. Effect of two factors' interactions on ABTS assay.

Source	Sum of squares	DF	Mean square	F	Probability
INTERACTIONS					
AB: Type of leaf and leaf granulometry	7.855	1	7.855	17.47	0.0024**
AC: Type of leaf and V/M ratio	2.477	1	2.477	5.51	0.0435*
AD: Type of leaf and Extraction time	0.400	2	0.200	0.44	0.6542 ns
BC: Leaf granulometry and V/M ratio	5.217	1	5.217	11.60	0.0078**
BD: Leaf granulometry and Extraction time	0.3188	2	0.159	0.35	0.7109 ns
CD: V/M ratio and Extraction time	0.033	2	0.017	0.04	0.9638 ns

Table S6. Effect of interactions of the two factors on the 1,8-cineole content.

Source	Sum of squares	DF	Mean square	F	Probability

**INTERACTIONS**					
AB: Type of leaf and leaf granulometry	26.818	1	26.818	2.67	0.1367 ns
AC: Type of leaf and V/M ratio	16.187	1	16.187	1.61	0.2361 ns
AD: Type of leaf and Extraction time	43.840	2	21.920	2.18	0.1687 ns
BC: Leaf granulometry and V/M ratio	94.129	1	94.129	9.37	0.0135*
BD: Leaf granulometry and Extraction time	8.188	2	4.094	0.41	0.6769 ns
CD: V/M ratio and Extraction time	3.406	2	1.703	0.17	0.8466 ns

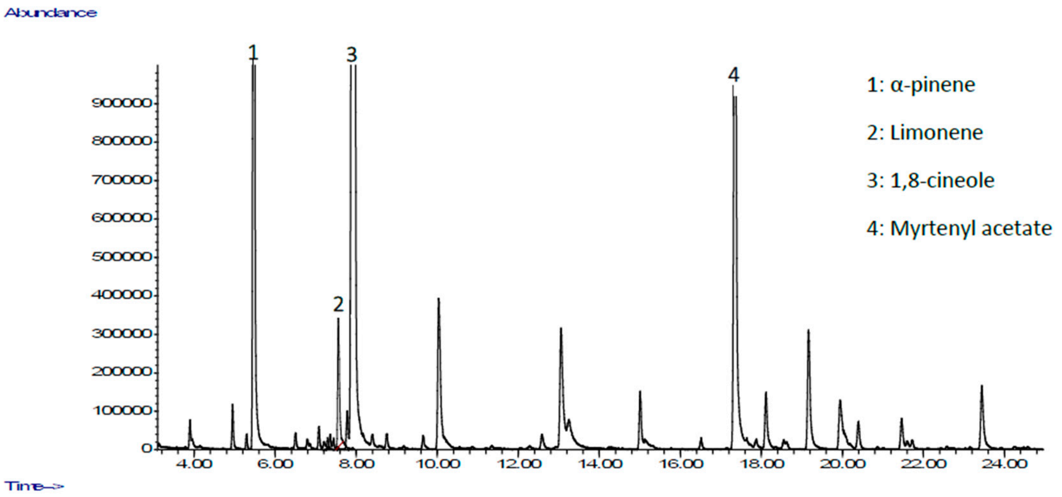

Figure S1. GC-MS chromatogram
